# Supplementary material for: Modeling glioblastoma heterogeneity as a dynamic network of cell states
Source: Mol Syst Biol. 2021 Sep 16;17(9):e10105. doi: 10.15252/msb.202010105 (PMC8444284; doi:10.15252/msb.202010105)
Supplement: Supplementary file 5 — Source Data for Figure 3 [file MSB-17-e10105-s001.zip › Figure3A_sourcedata/GSEA_3065/hallmarks_state4.GseaPreranked.1623416296068/HALLMARK_IL6_JAK_STAT3_SIGNALING.html]

Details for gene set HALLMARK\_IL6\_JAK\_STAT3\_SIGNALING[GSEA]

|  || Dataset | state4 |
| Phenotype | NoPhenotypeAvailable |
| Upregulated in class | na\_neg |
| GeneSet | HALLMARK\_IL6\_JAK\_STAT3\_SIGNALING |
| Enrichment Score (ES) | -0.44662142 |
| Normalized Enrichment Score (NES) | -1.2451062 |
| Nominal p-value | 0.20893142 |
| FDR q-value | 0.2524412 |
| FWER p-Value | 0.992 |
Table: GSEA Results Summary

  

Fig 1: Enrichment plot: HALLMARK\_IL6\_JAK\_STAT3\_SIGNALING      
 Profile of the Running ES Score & Positions of GeneSet Members on the Rank Ordered List

  

| PROBE | GENE SYMBOL | GENE\_TITLE | RANK IN GENE LIST | RANK METRIC SCORE | RUNNING ES | CORE ENRICHMENT || 1 | TNFRSF21 |  |  | 97 | 0.251 | 0.0959 | No |
| 2 | SOCS3 |  |  | 701 | 0.114 | 0.0497 | No |
| 3 | STAT2 |  |  | 824 | 0.102 | 0.0754 | No |
| 4 | GRB2 |  |  | 908 | 0.095 | 0.1043 | No |
| 5 | STAT1 |  |  | 1969 | 0.048 | -0.0444 | No |
| 6 | CSF1 |  |  | 2180 | 0.042 | -0.0594 | No |
| 7 | A2M |  |  | 2314 | 0.039 | -0.0635 | No |
| 8 | TNFRSF1A |  |  | 2315 | 0.039 | -0.0463 | No |
| 9 | TYK2 |  |  | 2621 | 0.032 | -0.0808 | No |
| 10 | CD44 |  |  | 2687 | 0.031 | -0.0774 | No |
| 11 | MYD88 |  |  | 2736 | 0.030 | -0.0719 | No |
| 12 | IL13RA1 |  |  | 2751 | 0.030 | -0.0610 | No |
| 13 | PTPN11 |  |  | 3030 | 0.024 | -0.0947 | No |
| 14 | IFNGR2 |  |  | 3147 | 0.022 | -0.1035 | No |
| 15 | IL6ST |  |  | 3218 | 0.021 | -0.1056 | No |
| 16 | TGFB1 |  |  | 3283 | 0.019 | -0.1076 | No |
| 17 | STAT3 |  |  | 3937 | -0.006 | -0.2096 | No |
| 18 | HAX1 |  |  | 3951 | -0.007 | -0.2088 | No |
| 19 | JUN |  |  | 3979 | -0.008 | -0.2094 | No |
| 20 | OSMR |  |  | 4176 | -0.020 | -0.2318 | No |
| 21 | IFNAR1 |  |  | 4576 | -0.049 | -0.2738 | No |
| 22 | TNFRSF1B |  |  | 5081 | -0.090 | -0.3148 | Yes |
| 23 | IRF1 |  |  | 5157 | -0.098 | -0.2835 | Yes |
| 24 | CD9 |  |  | 6176 | -0.378 | -0.2787 | Yes |
| 25 | TNFRSF12A |  |  | 6251 | -0.658 | 0.0019 | Yes |
Table: GSEA details [plain text format]

  

Fig 2: HALLMARK\_IL6\_JAK\_STAT3\_SIGNALING: Random ES distribution      
 Gene set null distribution of ES for **HALLMARK\_IL6\_JAK\_STAT3\_SIGNALING**
